# Dysregulation of the transcription factors SOX4, CBFB and SMARCC1 correlates with outcome of colorectal cancer

**DOI:** 10.1038/sj.bjc.6604884

**Published:** 2009-01-20

**Authors:** C L Andersen, L L Christensen, K Thorsen, T Schepeler, F B Sørensen, H W Verspaget, R Simon, M Kruhøffer, L A Aaltonen, S Laurberg, T F Ørntoft

**Affiliations:** 1 Molecular Diagnostic Laboratory, Department of Clinical Biochemistry, Aarhus University Hospital, Aarhus N DK8200, Denmark; 2 Department of Pathology, Aarhus University Hospital, Aarhus DK8000, Denmark; 3 Department of Gastroenterology and Hepatology, Leiden University Medical Center, Leiden 2300 RC, The Netherlands; 4 Department of Pathology, University Medical Center Hamburg-Eppendorf, Hamburg D-20246, Germany; 5 Department of Medical Genetics, Biomedicum, University of Helsinki, Helsinki FI 00290, Finland; 6 Department of Surgery, Aarhus University Hospital, Aarhus DK8000, Denmark

**Keywords:** gene expression, colorectal cancer, transcription factor, tissue microarray, clinical outcome

## Abstract

The aim of this study was to identify deregulated transcription factors (TFs) in colorectal cancer (CRC) and to evaluate their relation with the recurrence of stage II CRC and overall survival. Microarray-based transcript profiles of 20 normal mucosas and 424 CRC samples were used to identify 51 TFs displaying differential transcript levels between normal mucosa and CRC. For a subset of these we provide *in vitro* evidence that deregulation of the Wnt signalling pathway can lead to the alterations observed in tissues. Furthermore, in two independent cohorts of microsatellite-stable stage II cancers we found that high *SOX4* transcript levels correlated with recurrence (HR 2.7; 95% CI, 1.2–6.0; *P*=0.01). Analyses of ∼1000 stage I–III adenocarcinomas, by immunohistochemistry, revealed that patients with tumours displaying high levels of CBFB and SMARCC1 proteins had a significantly better overall survival rate (*P*=0.0001 and *P*=0.0275, respectively) than patients with low levels. Multivariate analyses revealed that a high CBFB protein level was an independent predictor of survival. In conclusion, several of the identified TFs seem to be involved in the progression of CRC.

Colorectal cancer (CRC) accounts for about 13% of all cancers and it is the second most common cause of cancer death in the western world (Parkin, 2001). Patients presenting with stage II CRC (no lymph node or distant metastases) have a 5-year survival of 75–80% when surgically treated. Surgical resection is highly effective in localised disease, but a significant proportion of stage II patients (20–25%) experiences recurrence and dies from the disease. Currently, it is not possible to accurately differentiate between groups of stage II patients with good and poor prognosis; consequently, there is an acute need for biomarkers that can distinguish between these two groups (Wang *et al*, 2004). Molecularly, CRC can be divided into two major subgroups: microsatellite-stable (MSS) and -unstable cancers (MSI). The two subgroups have a different clinical disease course, but are otherwise very similar. The mechanism responsible for MSI is known to be a failure of the DNA mismatch repair system. Recently, whole-genome transcriptional profiling studies have shown that there are profound transcriptional differences between MSS and MSI cancers (Giacomini *et al*, 2005; Kruhoffer *et al*, 2005). However, the causal mechanisms for these differences remain unclear. The biological and clinical behaviour of CRC is affected by multiple molecular pathways controlling cellular processes like differentiation, migration, replication, DNA repair, proliferation and apoptosis (Sancho *et al*, 2004); one particularly well-studied pathway is the Wnt pathway (de Lau *et al*, 2007). Ultimately, the transcriptional responses mediated by these pathways are controlled and exercised by transcription factors (TFs). In normal tissue, tight control of the levels of TFs is crucial to maintain tissue homoeostasis and, hence, many TFs have been found to be oncogenic when their expression is deregulated or when their functionality is altered through fusion with other genes (Look, 1997). An improved understanding of which TFs affect cancer biology may lead to improved ability to predict clinical outcome and discovery of novel therapeutic strategies.

In this study, we used microarray expression profiling to identify 51 TFs significantly deregulated in CRC. The list included not only several TFs known to be deregulated in CRC but also many for which this information was novel. The expression of the 51 TFs was further investigated in relation to clinically important CRC subgroups. These analyses showed that 12 TFs were differentially expressed between the MSS and MSI tumour subgroups, that a high transcript level of one TF (*SOX4*) was significantly associated with poor outcome of stage II MSS cancer and that high protein levels of CBFB and SMARCC1 were correlated with increased overall survival of CRC. In relation to the mechanisms causing the TF deregulation, we provide evidence that deregulation of the Wnt pathway influences the expression level of ∼20% of the 51 TFs.

## Materials and methods

### Patients and samples

Colorectal samples from a total of 444 patients, comprising 20 adjacent normal mucosa and 424 primary colorectal carcinomas, were used for array-based transcriptional profiling. The 20 adjacent normal mucosa samples were all collected from non-cancer areas located orally for the tumour. None of the patients had received preoperative chemo- and/or radiotherapy. The samples were organised into two data sets. The first data set termed ‘HG_U133A’ is a subset of a previously published set of transcription profiles (Kruhoffer *et al*, 2005) and consisted of 10 adjacent normal mucosa and 80 colon cancer samples, comprising 53 stage II and 27 stage III cancers with a median age at diagnosis of 68.5 years (range 36–87 years). The MSS/MSI status was known for 76 of the 80 cancers (25 MSI and 51 MSS). The second data set termed ‘HG_U133 plus 2.0’ consisted of 10 adjacent normal mucosa and 344 cancer samples (288 located in the colon and 56 in the rectum). The cancer samples comprised 23 stage I, 287 stage II, 22 stage III and 12 stage IV cancers with a median age at diagnosis of 70 years (range 29–94 years). The MSS/MSI status was known for 331 of the 344 cancers (73 MSI and 258 MSS).

For the ‘HG_U133 plus 2.0’ set, time to recurrence information was available for 326 of the 332 stage I–III cancers. The median duration of follow-up was 59.8 months (range 12.8–145.6 months) for the 274 patients without recurrence as the first event and 18.7 months (range 1.2–78.7 months) for the 52 patients with distant recurrence as the first event.

Detailed lists of the clinicopathological data available for the 444 samples can be found in Supplementary Tables 1 and 2. Informed consent was obtained from all patients according to local ethical regulations, and all studies were approved by local ethical committees according to the Helsinki Declaration.

Three human colorectal carcinoma tissue microarrays (TMAs) were used for immunohistochemistry (IHC) analysis. A commercial TMA (catalogue no. COCA 912-5-OL; BioCat, Heidelberg, Germany) for the evaluation of associations of the normal adenoma-carcinoma sequence with 71 colorectal specimens (10 normal colon mucosa, 10 adenoma, 40 stage I–IV adenocarcinomas and 11 liver metastasis), a custom-made stage II TMA containing 51 stage II adenocarcinomas and 50 normal mucosa specimens, and a large custom-made TMA containing CRC biopsies from 1461 patients (Went *et al*, 2006). The latter TMA was used for survival analyses. Therefore, patients with missing values for any of the following parameters gender, age at surgery, tumour location, TNM stage and survival time were excluded. Likewise, patients who died of surgical complications (dead within 30 days of surgery) were excluded, leaving 1283 out of 1461 patients for survival analysis. Their median age at diagnosis was 71 years (range 30–96 years). The 1283 cancers comprised 177 stage I, 492 stage II and 614 stage III cancers. The median duration of follow-up was 52.4 months (range 1–152 months). Data on post-surgery radio- or chemotherapy were not available. Detailed clinicopathological data of the TMA specimens are given in Supplementary Tables 3A, 4 and 5. The WHO/UICC-TNM staging system was used for staging of the adenocarcinomas.

### Labelling, hybridisation, normalisation and statistical analysis of microarrays

Labelling of RNA, hybridisation and scanning of HG_U133 plus 2.0 and Human Exon 1.0 ST arrays (Affymetrix, Santa Clara, CA, USA) were performed as described earlier (Dyrskjot, 2003; Thorsen *et al*, 2008). The Human Genome U133A GeneChip array-based transcription profiles used in this study is a subset of a previously published set of transcription profiles (Kruhoffer *et al*, 2005). GC-content corrected robust multiarray analysis (GC-RMA) normalisation of the U133 plus 2.0 arrays and iterPLIER normalisation of the Exon 1.0 ST arrays were performed using the software packages ArrayAssist (Stratagene, La Jolla, CA, USA) and Expression Console v1.1 (Affymetrix), respectively. Differentially expressed genes were identified using a Benjamini and Hockberg-corrected ‘Two class unpaired’ statistical tests.

### Approach for the identification of transcriptionally deregulated TFs

A list of candidate TFs was generated by querying the Gene Ontology (GO) database for genes linked to the GO term ‘transcription’. In this way, the Affymetrix HG_U133A array was found to contain probe sets for 1026 potential TFs (1026 unique Entrez Gene IDs). To identify if any of these candidate TFs were deregulated in CRC compared with normal mucosa, we used the two independent sets of array-based transcriptional profiles described in the ‘Patients and samples’ section. The candidate TFs with an average numerical log 2 fold change of at least 1 (1 on log 2 scale, corresponds to a fold change of 2 on a linear scale) that were significantly deregulated (*P*<10^−4^ after Benjamini and Hochberg correction) in the ‘HG_U133A’ set (80 cancers/10 normals) were taken further for validation in the second larger and independent set ‘HG_U133 plus 2.0’ (344 cancers/10 normals). Transcription factor deregulation was considered significant if a fold change of at least 1 (in the same direction as in the ‘HG_U133A’ set) with a *P*-value lower than 10^−4^ was obtained in the validation set.

### Real-time RT–PCR

Quantitative real-time RT–PCR (qRT–PCR) was performed on a 7900HT Real-Time PCR System (Applied Biosystems, Foster City, CA, USA) using the relevant (TaqMan or SYBR Green) Master Mix (Applied Biosystems). Predesigned TaqMan assays were used to measure *RB1* (Hs01078066_m1) and *E2F3* (Hs00605457_m1) (Applied Biosystems). For normalisation, the gene *Ubiquitin C* (*UBC*) was employed. We have demonstrated earlier the suitability of *UBC* as a normalisation gene for analysis of normal mucosa and CRC specimen sample sets (Andersen *et al*, 2004). The UBC primer sequences have been published earlier (Andersen *et al*, 2004). cDNA was generated using the Superscript™ cDNA synthesis kit (Invitrogen, Carlsbad, CA, USA) with random nonamer primers and RNAse inhibitor (Ambion, Austin, TX, USA). Each measurement was performed in triplicate, and no-template controls were included for each assay. Relative expression values were obtained using a four-point 10-fold dilution curve. The dilution curve was created using a cDNA pool of each of the test cDNAs.

### Cell culturing

Dominant-negative TCF1 (dnTCF1)-inducible LS174T-derived cell lines have earlier been described (van de Wetering *et al*, 2002) and were a kind gift from Dr Hans Clevers (The Hubrecht Laboratory, Utrecht, The Netherlands). The cell lines were cultured and dnTCF1 induced by doxycycline as described earlier (Schepeler *et al*, 2007). The cell lines were free from mycoplasm contamination as verified by the MycoSensor™ PCR assay kit (Stratagene) according to the manufacturer's instructions. Cells were harvested at 0 and 12 h after doxycycline induction. mRNA for array-based transcript profiling was extracted using the GenElute™ Mammalian total RNA miniprep kit (Sigma-Aldrich, Brandlay, Denmark).

### Antibodies

Mouse monoclonal antibodies usable for IHC and immunoblotting were available for the TFs CBFB (catalogue no. ab11921 (clone 141,4,1); Abcam, Cambridge, UK), E2F3 (catalogue no. 05-551; Upstate, Charlottesville, VA, USA) and SMARCC1 (catalogue no. sc-32763 (DXD7); Santa Cruz Biotechnology, Santa Cruz, CA, USA). A rabbit polyclonal anti-Sox4 antibody (catalogue no. AB5803; Chemicon International Inc., Temecula, CA, USA) usable for IHC was also available.

The specificity of the E2F3 and SMARCC1 antibodies was validated by immunoblot analysis of COS7 cells transiently transfected with expression plasmids containing full-length cDNAs (Supplementary Figures 1A and B). The specificity of the CBFB antibody was validated by immunoblot analysis of normal mucosa and colon cancer, which yielded bands corresponding to the known CBFB isoforms (Supplementary Figure 1C). The SOX4 antibody did not work in western blot analysis. However, we have earlier used IHC to investigate SOX4 protein expression in bladder cancer (Aaboe *et al*, 2006). Hence, bladder cancer specimens were used as positive controls for the SOX4 antibody. A pan-leukocyte marker, CD45, in the form of the mouse monoclonal anti-CD45 antibody (catalogue no. MS-240-P1; Neomarkers/Lab Visions corp., Fremont, CA, USA) was used to identify infiltrating lymphocytes (Woodford-Thomas and Thomas, 1993).

### Immunohistochemistry

Standard indirect staining procedures were used for IHC (Went *et al*, 2006). In brief, paraffin was removed followed by blocking of endogenous peroxidase activity, heat-induced epitope demasking and blocking to prevent unspecific binding. Then primary antibodies were applied; the following antibodies were used, anti-CBFB (1 : 1800), anti-SMARCC1 (1 : 150), anti-E2F3 (1 : 200), anti-SOX4 (1 : 800) and anti-CD45 (1 : 600). Detection was performed using the Envision system (DakoCytomation, Glostrup, Denmark). Finally, the sections were counterstained with haematoxylin.

Semiquantitative scoring of intensity (negative, 0; weak, 1;and strong, 2) and fraction of positive cancer cells (negative, 0; less than half, 1; and more than half, 2) was undertaken independently by two investigators. Nuclear and cytoplasmic staining was scored separately. Tissue microarray cores that were missing or contained fewer than 10 cancer cells or demonstrated significant artefacts were not scored. Kappa statistics was used to evaluate the agreement between the observers. In all scoring categories (intensity, fraction, nuclear and cytoplasmic) a good-to-very good inter-observer agreement was obtained for all investigated TFs (Kappa statistic values ranging from 0.73 to 1.00, median 0.90). The scores were converted into simple categories of negative, weak and strong staining using the following formula: negative: intensity 0 and fraction of positive cells 0; weak: intensity 1 and fractions 1 and 2, and intensity 2 and fraction 1; strong: intensity 2 and fraction 2.

### Statistical analysis

The software used for statistical analysis was STATA 9.2 (StataCorp, College Station, TX, USA). Fisher's exact and *χ*^2^ tests were used to compare TF expression and clinical and morphological tumour characteristics.

For each individual IHC score (nuclear and cytoplasmic), survival analysis was performed. For simplicity, the nuclear and cytoplasmic scores were combined when they showed the same statistical trend. The scores were combined using the following formula: negative: both scores were negative; weak: if neither score was strong and one or both were weak; and strong: one or both scores were strong. Survival curves were plotted according to Kaplan–Meier. Univariate analysis was performed using the log-rank test or the Cox proportional hazards model. Multivariate analysis was performed using the Cox proportional hazards regression model. *P*-values less than 0.05 were considered significant.

## Results

### Identification of deregulated TF transcripts in colorectal cancer

By comparing transcript profiles of normal mucosa and CRC samples in two independent sample sets, covering a total of 20 normal mucosa and 424 adenocarcinoma samples, we identified 51 TFs that showed a significant (*P*<10^−4^, Benjamini and Hochberg corrected) fold change (of at least 2) from normal to cancer in both data sets. Fourteen of these TFs were downregulated and 37 upregulated (Table 1). The list included not only several TFs known to be deregulated in CRC, for example *c-MYC* (He *et al*, 1998) and *SOX9* (Blache *et al*, 2004), but also many for which this information was novel for example, CBFB and SMARCC1. Below we have validated four of the latter TFs, the others will have to be validated in future studies.

### Transcriptional analysis of E2F3 and RB1

One of the 51 deregulated TFs was *E2F3*, which belongs to the family of E2F TFs. The E2F factors and the tumour suppressor RB1 constitute the key players in the RB/E2F pathway, a critical regulator of G1/S cell cycle transition and hence proliferation. Mutational inactivation of this checkpoint has been associated with many cancers, but not CRC (Nevins, 2001). Our transcript profiling data from the ‘HG_U133 plus 2.0’ data set (10 normal mucosa samples and 344 cancers) showed that the average *E2F3* transcript level was 1.67-fold (log 2 scale) higher in cancer than in normal mucosa (Table 1), whereas *RB1* remained unchanged (*P*=0.26, two class unpaired test Benjamini and Hockberg corrected). A comparison of the *RB1/E2F3* transcript ratios revealed a significant reduction of −1.97 fold (log 2 scale) from normal mucosa to cancer (*P*=8.96 × 10^−8^, Student's *t*-test). A similar reduction was found in the ‘HG_U133A’ data set (10 normal mucosa samples and 80 cancers) with the average *RB1/E2F3* ratio dropping −0.63-fold (log 2 scale) from normal mucosa to cancer (*P*=0.019, Student's *t*-test). To validate that the reduction was not an artefact of measuring the transcript levels using array technology, we selected 60 samples (10 normals and 50 randomly selected cancers) from the HG_U133 plus data set for qRT–PCR analysis. Quantitative RT–PCR confirmed that the *E2F3* transcript level was increased in cancer (2.22-fold, log 2 scale; *P*=1.26 × 10^−7^, Student's *t*-test) and that the average *RB1/E2F3* ratio was reduced by −0.87-fold (log 2 scale) from normal mucosa to cancer (*P*=3.52 × 10^−6^, Student's *t*-test).

**Figure 1:**
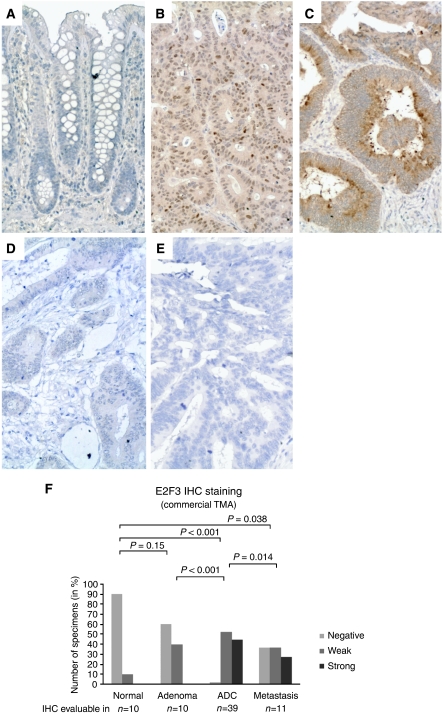
E2F3 expression detected by immunohistochemistry. E2F3 IHC analysis of the commercial TMA, COCA 912-5-OL, containing normal mucosa as well as benign and malignant colorectal specimens demonstrated that although E2F3 was not expressed by normal epithelial cells (**A**) E2F3 was found *de novo* synthesised by the majority of the investigated neoplastic tissues (**B** and **C**). The subcellular localisation of the *de novo* synthesised E2F3 protein was in some tumours found to be primarily nuclear (**B**) and cytoplasmic in others (**C**). Adenocarcinomas with no E2F3 staining (negative) were also observed, though only rarely (**D**). These were very similar to the ‘no primary’ antibody negative control (**E**). The frequency and intensity of E2F3 protein expression (combining nuclear and cytoplasmic staining) in adenoma, adenocarcinoma and metastasis samples were significantly higher than in normal mucosa (**F**). The same was the case when nuclear and cytoplasmic staining was evaluated individually (data not shown). The E2F3 IHC staining was evaluable and scored in 70 of the 71 tissue cores in the commercial TMA. *P*-values correspond to Fisher's exact tests. ADC=adenocarcinoma. All images are × 20. Staining: brown, E2F3; blue, haematoxylin counterstain.

E2F3 IHC analysis of a commercial TMA containing normal mucosa as well as benign and malignant colorectal specimens revealed that E2F3 was significantly higher expressed also at the protein level in the majority of cancers compared with normal mucosa (Figure 1A–F). The data also indicated that the E2F3 protein level might be associated with tumour progression as a significant difference was observed between adenocarcinomas and metastases (Figure 1F). To investigate if the E2F3 protein level was associated with TNM stage and overall survival, IHC was applied to a large custom-made TMA with stage I–III adenocarcinomas, for which follow-up information was available. A borderline-significant association was found for TNM stage (*P*=0.066, Fisher's exact test; Supplementary Table 3B). This resulted from a minor but significant shift from strong to weak E2F3 staining from stage I to II (*P*=0.011, Fisher's exact test), which was not repeated from stage II to III (*P*=0.593, Fisher's exact test). To test if the E2F3 expression level followed a stage-specific trend, we applied a non-parametric test for trend (an extension of the Wilcoxon rank-sum test (Cuzick, 1985)) that did not reach significance (*P*=0.183). Taken together the data indicate that there is no tight association between the E2F3 protein level and TNM stage. Likewise, our survival analysis did not identify any relationship between the E2F3 protein level and overall survival (data not shown). In Supplementary Table 3B, correlations of E2F3 with other histopathological parameters are shown.

### TF expression in microsatellite stable and unstable colon cancers

Two major molecular subgroups of CRC have been discerned – MSS and MSI cancers. As the cellular processes responsible for both the similarities and differences between the MSI and MSS subgroups are likely to be regulated by TFs, we wanted to investigate the expression patterns of the deregulated TFs in MSS and MSI cancers. To avoid tumour site from becoming a confounding factor, the analysis was restricted to colon cancers. This was motivated by the observation that MSI occurred more frequently in colon (24%) than rectal cancer (12.5%) in our ‘HG_U133 plus 2.0’ sample set. Similar observations have been reported earlier (Ikeda *et al*, 2001). Naturally, only colon cancers for which the MSS/MSI status was known were included. Hence, from the ‘HG_U133A’ sample set, 76 of 80 colon cancers (51 MSS and 25 MSI) were included and from the ‘HG_U133 plus 2.0’ data set, 275 of 288 colon cancers (209 MSS and 66 MSI). To exclude that TNM stage distribution was a confounding factor, we used Fisher's exact tests to compare the distribution of TNM stages in the MSS and MSI subgroups. No significant differences were observed (*P*>0.05).

Comparison of the MSS and MSI subgroups revealed that 12 of the 51 TFs were differentially expressed (*P*<0.05, Benjamini and Hockberg-corrected ‘Two class unpaired’ test) in both data sets (Table 2 and Supplementary Table 6). Ten TFs showed lower, and two higher, expression in MSI relative to MSS cancer. A comparison of the expression levels of the 12 TFs in normal mucosa and MSS and MSI cancers revealed that *FOXA2* was significantly (*P*<0.01) deregulated in MSS but not MSI cancers explaining why it was found to be differentially expressed between MSS and MSI. The remaining 11 TFs were all deregulated (normal to cancer) in both cancer subgroups (in the same direction), but the degree of deregulation differed explaining why they were found to be differentially expressed between MSS and MSI (data not shown).

### TF expression in relation to recurrence of stage II CRC

Deregulation of TFs may lead to the development of cancer and progression of disease (Look, 1997; Nesbit *et al*, 1999). Along these lines we speculated if the transcriptional level of any of the identified TFs (Table 1) might be involved in determining the metastatic capacity of primary tumours. To test this hypothesis, we investigated transcriptional profiles of two series of stage II CRCs (an MSS and an MSI series), which were selected from our set of 287 stage II ‘HG_U133 plus 2.0’ transcription profiles. Twenty-seven cases were excluded based on the following criteria: missing MSS/MSI status (*n*=11), missing follow-up (*n*=6), follow-up <40 months (*n*=1) or distant recurrence to sites other than liver or lungs (*n*=9). The remaining 260 cases were divided into two series based on their MSS/MSI status. None of the included cases had received adjuvant therapy. The stage II MSS series consisted of 173 non-recurrent cases with a median duration of follow-up of 59.8 months (range 43.3–145.6 months) and 22 recurrent cases, that is histologically verified liver and/or lung metastases, with a median duration of follow-up of 16.7 months (range 4.9–78.7 months). Similarly, the MSI series consisted of 56 non-recurrent cases with a median duration of follow-up of 59.8 months (range 51.1–109.2 months) and nine recurrent cases with a median duration of follow-up of 30.0 months (range 1.2–65.9 months). Unpaired *t*-tests indicated that three of the 51 TFs were significantly associated (*P*<0.05) with recurrence of stage II MSS cancer and 13 with recurrence of MSI stage II cancer (Table 2 and Supplementary Table 7). From a recent report we extracted Affymetrix HG_U133A transcriptional profiles of 25 non-recurrence and 25 recurrence (liver metastases) stage II MSS colon cancers (Barrier *et al*, 2006). By analysing this independent data set one of the three significant TFs (*SOX4*) from our MSS series was validated (*P*=0.0039, unpaired *t*-test). The other two TFs did not reach significance. To our knowledge, no publicly available data set of stage II MSI CRCs with follow-up information exists. Hence, we have not been able to validate the 13 candidate markers of recurrence of stage II MSI cancer.

**Figure 2:**
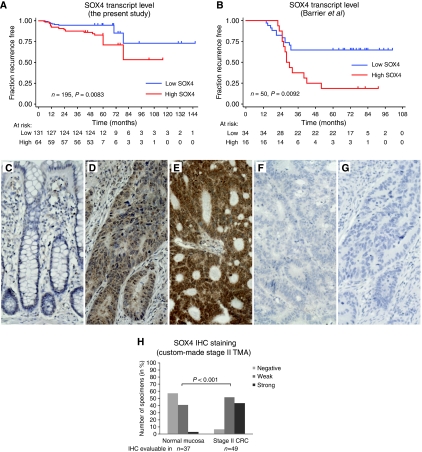
Analysis of SOX4 transcript and protein expression levels. Shown are censored Kaplan–Meier curves for recurrence-free survival of MSS stage II CRC according to the transcriptional expression level of *SOX4* as measured by microarray expression profiling in this study (**A**) and the study by Barrier *et al* (2006) (**B**). The *P*-values correspond to the log-rank test. To investigate whether the SOX4 protein expression level changed from normal mucosa to adenocarcinoma, IHC was applied to a custom-made stage II TMA. The TMA tissue cores were not scored if they were missing, or contained fewer than 10 cancer cells, or demonstrated significant tissue or IHC staining artefacts. Hence, only 49 of the 51 adenocarcinoma and 37 of the 49 normal mucosa cores in the TMA were scored. In the majority of the normal mucosa samples, SOX4 was either not (**C**) or only weakly expressed. By contrast, a strong SOX4 staining was observed in more than 40% of the stage II adenocarcinomas. Often, the SOX4 protein was localised both in the cytoplasm and in the nucleus of the cancer cells. In some adenocarcinomas, the SOX4 staining was strongest in the nucleus (**D**), whereas in others, the cytoplasm and the nucleus were stained equally strong (**E**). Adenocarcinomas with no SOX4 staining (negative) were also observed, though only rarely (**F**). These were highly similar to the ‘no primary’ antibody negative control (**G**). The distribution of SOX4 IHC scores (combined nuclear and cytoplasmic staining) showed that both the frequency and the intensity of SOX4 staining were increased in the adenocarcinomas compared with those in the normal mucosas (**H**). The same was the case when the nuclear and cytoplasmic IHC scores were analysed individually (data not shown). The *P*-values were calculated using the *χ*^2^ test. All IHC images are × 20. Staining: brown, SOX4; blue, haematoxylin counterstain.

Next we investigated the association between the *SOX4* transcript level and recurrence-free survival. To enable survival analysis, the *SOX4* expression data were dichotomised with the highest tertile classified as high and the lower two tertiles classified as low. This cutoff was set based on associations within the MSS stage II CRC cohort of this study before analysing the validation cohort from Barrier *et al* (2006). As expected, the patients having an *SOX4* expression level in the highest tertile experienced a significantly higher incidence of recurrence (*P*=0.005, *χ*^2^ test) and a shorter recurrence-free survival (Figure 2A, log-rank test *P*=0.0083 and univariate cox regression analysis *P*=0.01; hazard ratio 2.0; 95% CI, 1.18–3.51). Notably, this association was only observed in our MSS series (Table 2), consistent with *SOX4* being differentially expressed between MSS and MSI tumours (Table 2). In the validation data set from Barrier *et al* (2006)*, SOX4* expression in the upper tertile was also associated with a significantly higher incidence (*P*=0.005, Fisher's exact test) of recurrence and a shorter recurrence-free survival (log-rank test *P*=0.0092 and univariate cox regression analysis *P*=0.01; hazard ratio 2.7; 95% CI, 1.2–6.0). Recurrence-free survival as a function of *SOX4* expression is plotted in Figure 2B. Subsequently, we wanted to investigate if the *SOX4* transcripts observed in cancer were also translated into protein. To address this question we returned to our comparisons of normal mucosa and adenocarcinomas, which demonstrated increased transcript levels in the adenocarcinomas (Table 1). We then performed a similar analysis at the protein level by applying SOX4 IHC to a custom-made stage II TMA. The IHC staining was evaluable in 37 normal mucosa and 49 adenocarcinoma tissue cores (all from patients independent from the cohort investigated by transcriptional profiling). In agreement with the transcript analysis, IHC revealed that both the frequency and the expression level of SOX4 protein were generally increased in adenocarcinomas, *P*<0.001 Fisher's exact test (Figure 2C–H). Furthermore, the IHC analysis demonstrated that the SOX4 protein found in the tumours was primarily of cancer cell origin (Figure 2D and E). We did not have recurrence nor survival information for the patients in the stage II TMA. Consequently, we could not evaluate if the SOX4 protein level could also predict recurrence of stage II CRC. Nor could we analyse if it was associated with survival. However, this would be interesting to investigate in a future study.

### CBFB and SMARCC1 protein expression and association with overall survival

**Figure 3:**
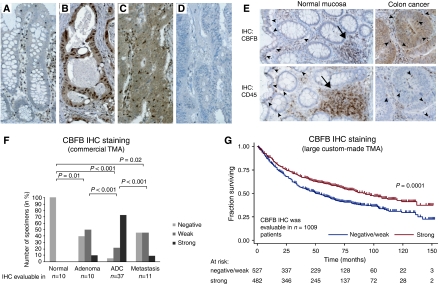
CBFB protein expression and the relation to survival. The CBFB protein expression was investigated by IHC in normal mucosa, adenoma, adenocarcinoma and metastases using the commercial TMA COCA 912-5-OL. Tissue microarray tissue cores were not scored if they were missing, or contained fewer than 10 cancer cells, or demonstrated significant tissue or staining artefacts. Hence, of the 71 tissue cores in the commercial TMA, 68 were scored for CBFB expression. The IHC analysis showed that whereas CBFB was not expressed by normal epithelial cells (**A**) CBFB was found *de novo* synthesised by the majority of the investigated neoplastic tissues (**B** and **C**). The subcellular localisation of the *de novo* synthesised CBFB protein was in some tumours found to be primarily nuclear (**B**) and cytoplasmic in others (**C**). Adenocarcinomas with no CBFB staining (negative) were also observed (**D**). Often small intensely staining cells were seen in the stroma and infiltrating both the normal epithelia (**A**) and the cancer cells (**C**). Comparison of sections cut from the same biopsies and stained with CBFB and CD45 (a pan-leukocyte marker) indicated that the small cells with intense CBFB staining represent infiltrating leukocytes (**E**). The distribution of the CBFB IHC scores in normal mucosa, adenoma, adenocarcinoma and metastases illustrates that CBFB is *de novo* synthesised in neoplastic tissue and that the frequency and intensity of the staining (combined cytoplasmic and nuclear staining) increases from adenoma to adenocarcinoma, and then drops back down again in metastases (**F**). Similar results were reached when the cytoplasmic and nuclear staining were analysed individually (data not shown). *P*-values correspond to Fisher's exact tests. To investigate if the CBFB protein level was correlated with overall survival, IHC was applied to a large custom-made TMA containing adenocarcinoma tissue cores from 1283 patients with available follow-up information. The CBFB IHC staining was evaluable in 1009 of these patients. Shown in (**G**) are censored Kaplan–Meier curves as a function of the CBFB IHC scores (nuclear and cytoplasmic scores combined). The individual survival curves for patients with negative and weak IHC scores were very similar, and they were therefore treated as one group. Similar results were reached when the cytoplasmic and nuclear staining were analysed individually (data not shown). The *P*-value corresponds to the log-rank test comparing the survival curves. See Supplementary Table 3A for a summary of the clinical characteristics and follow-up information available for the 1009 patients. See Supplementary Table 3C for the distribution of the 1009 IHC scores and their correlations with the available clinicopathological parameters. Arrow heads: infiltrating lymphocytes. Arrows: lymphoid nodules. All images are × 20. Staining: brown, CBFB or CD45 as indicated; blue, haematoxylin counterstain.

**Figure 4:**
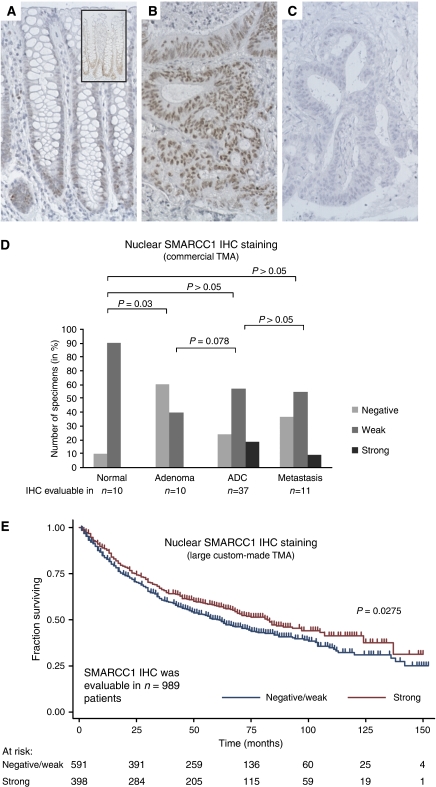
SMARCC1 protein expression and the relation to survival. The SMARCC1 protein expression was investigated by IHC in normal mucosa, adenoma, adenocarcinoma and metastases using the commercial TMA COCA 912-5-OL. Tissue microarray tissue cores were not scored if they were missing, or contained fewer than 10 cancer cells or demonstrated significant tissue or staining artefacts. Hence, of the 71 tissue cores in the commercial TMA, 68 were scored for SMARCC1 expression. The IHC analysis showed that in normal colon mucosa, SMARCC1 was weakly expressed in the lower third of the crypts of (**A**). The inset in (**A**) represents an IHC analysis of the same specimen but without the counterstain, making the SMARCC1 staining at the bottom of the crypts stand out more clearly. In contrast to the normal mucosa, the SMARCC1 staining seen in the neoplastic tissues was often more intense and uniformly distributed (**B**). Neoplastic tissues with no SMARCC1 staining (negative) were also observed (**C**). The subcellular localisation of the SMARCC1 protein was in both the normal epithelial cells and in the neoplastic cells restricted to the nucleus (**A** and **B**). As SMARCC1 was already expressed by the normal mucosa, comparisons of the SMARCC1 IHC scores in normal mucosa, adenocarcinoma and metastases revealed no significant differences (**D**). *P*-values correspond to Fisher's exact tests. To investigate if the SMARCC1 protein level was correlated with overall survival, IHC was applied to a large custom-made TMA containing adenocarcinoma tissue cores from 1283 patients with available follow-up information. The SMARCC1 IHC staining was evaluable in 989 of these patients. Shown in (**E**) are censored Kaplan–Meier survival curves as a function of the SMARCC1 IHC scores. The individual survival curves for patients with negative and weak IHC scores were very similar and they were therefore treated as one group. The *P-*value corresponds to the log-rank test comparing the survival curves. See Supplementary Table 3A for a summary of the clinical characteristics and follow-up information available for the 989 patients. See Supplementary Table 3D for the distribution of the 989 IHC scores and their correlations with the available clinicopathological parameters. All images are × 20. Staining: brown, SMARCC1; blue, haematoxylin counterstain.

Of the TFs not reported earlier to be associated with CRC, we selected CBFB and SMARCC1 for IHC analysis based on the availability of antibodies for which the specificity could be validated in western blot analyses. To investigate CBFB and SMARCC1 protein expression in normal and neoplastic tissues, IHC was applied to a commercial TMA (COCA 912-5-OL) containing tissue biopsies covering normal mucosa, adenoma, adenocarcinoma and liver metastases. No CBFB protein expression was observed in normal epithelia (Figure 3A). SMARCC1 was found to be weakly expressed in the basal half of the normal colonic crypts (Figure 4A). CBFB was observed both in the nucleus and in the cytoplasm of the neoplastic cells, whereas SMARCC1 was observed only in the nucleus in both normal and neoplastic cells (Figure 3B and C and 4B, respectively). A comparison of IHC staining for CBFB and CD45 (a pan-leukocyte marker) indicated that CBFB was also expressed by a subset of stromal cells, in particular lymphocytes, including lymphocytes infiltrating both normal epithelia and cancer (Figure 3E). We then compared the staining frequencies observed in normal mucosa, adenoma, adenocarcinoma and liver metastasis. For simplicity, the nuclear and cytoplasmic CBFB IHC scores were combined to a single CBFB score in these analyses. The frequency and intensity of CBFB expression increased significantly (*P*=0.01, Fisher's exact test) from normal mucosa to adenoma and again from adenoma to adenocarcinoma (*P*<0.001, Fisher's exact test). By contrast, the staining frequency in liver metastases was significantly lower than that in adenocarcinomas (*P*<0.001, Fisher's exact test) (Figure 3F). Overall, the IHC analysis indicated that the CBFB protein level was increased in tumours compared with normal mucosa. This was in agreement with the transcriptional data presented above.

SMARCC1 showed weak staining in the basal half of the normal crypts in 9 of 10 specimens. In contrast, only 4 of 10 adenomas showed staining. Contrary to the normal mucosa, the adenomas were uniformly stained. The frequency and intensity of SMARCC1 staining increased borderline significantly (*P*=0.078, Fisher's exact test) from adenoma to adenocarcinoma, but not from adenocarcinoma to liver metastases (Figure 4D). Generally, the staining intensities observed in the neoplastic tissues were more intense than in the normal mucosa (Figure 4A and B). This combined with a more uniform staining pattern indicated that the overall SMARCC1 protein level was generally higher in neoplastic tissue than in normal mucosa. This is in accordance with the SMARCC1 transcriptional data presented above.

To investigate the possible associations between the protein levels of CBFB and SMARCC1 and the TNM stages and overall survival, IHC was applied to a large custom-made TMA with stage I–III adenocarcinomas for which follow-up information was available. For both TFs, the frequency of strong staining appeared to decrease with increasing TNM stage; however, the tendencies were insignificant in both instances (*P*>0.05, Fisher's exact test; Supplementary Tables 3C and D). When a less-stringent test for trend (an extension of the Wilcoxon rank-sum test (Cuzick, 1985)) was applied, the tendencies were significant; *P*=0.029 and *P*=0.026 for CBFB and SMARCC1, respectively (Supplementary Tables 3C and D). Next, the possible correlations between CBFB and SMARCC1 protein levels and overall survival were investigated. Kaplan–Meier survival curves are shown in Figures 3G and 4E. Patients with tumours showing strong staining had a significantly longer survival rate than patients with negative or weak scoring tumours (log-rank test; CBFB *P*=0.0001; SMARCC1 *P*=0.0275). As both CBFB and SMARCC1 had shown weak correlations with TNM stage, the Kaplan–Meier survival analyses were repeated and this time was stratified by TNM stage (Supplementary Figure 2). Bivariate Cox proportional hazard regression analyses showed that while the CBFB protein level remained a significant prognostic factor after stratification for TNM stage (*P*=0.002), the SMARCC1 protein level was only borderline significant (*P*=0.073) (Supplementary Figure 2). To elaborate the analysis, it was extended from a bi- to a multivariate analysis including all the available variables that were significant in univariate analysis (i.e. age at surgery, sex, differentiation grade, TNM stage, CBFB staining and SMARCC1 staining). The multivariate analysis showed that while the CBFB protein level remained a significant prognostic factor after stratifying for all other available risk parameters (*P*=0.011) the SMARCC1 protein level did not (Table 3).

### TF expression in relation to Wnt signalling

The Wnt-signalling pathway is aberrantly activated in the majority of CRCs and contributes directly to malignant transformation by influencing the transcript levels of numerous genes (van de Wetering *et al*, 2002; de Lau *et al*, 2007). To investigate if changes in the activity of the Wnt-signalling pathway could influence the expression level of any of the 51 TFs we utilised a colon carcinoma model system based on LS174T cells. The model system was engineered so that Wnt signalling mediated by *β*-catenin/TCF could be abrogated on demand by induction of dnTCF1 (van de Wetering *et al*, 2002), a naturally occurring inhibitor of Wnt signalling (Waterman, 2004). Using two different microarray platforms, the transcript levels of all 51 TFs were measured at 0 and 12 h after dnTCF1 induction. Induction was validated directly and indirectly by qRT–PCR. Directly, by demonstrating increased levels of dnTCF1 transcript, and indirectly, by demonstrating downregulation of c-Myc, a well-known target of the inhibited *β*-catenin/TCF complex (Supplementary Figure 3). Both microarray platforms consistently showed deregulation of 11 TFs (seven downregulated, including c-MYC, and four upregulated) in cells in which Wnt signalling was inactivated (Table 2 and Supplementary Table 8). In the majority of CRCs, Wnt signalling is abnormally active; consequently, we would expect genes downregulated by Wnt inactivation to be upregulated in cancer and vice versa for genes upregulated by Wnt inactivation. For 9 of the 11 Wnt-regulated TFs, this was exactly what we observed (Supplementary Table 8). The seven TFs downregulated by Wnt inactivation were all upregulated in cancer, and two of the four TFs upregulated by Wnt inactivation (*HIS1* and *SSBP2*) were downregulated in cancer. The expression levels of the remaining 40 TFs did not consistently follow Wnt-signalling activity. Hence, for ∼20% of the 51 TFs a likely cause for the observed deregulation in cancer is abnormal Wnt signalling.

## Discussion

This study reports the results from a thorough analysis of TF expression changes in CRC. Our analysis of array-based transcriptional profiles of 20 normal mucosa and 424 CRC samples indicated that 51 TFs were significantly deregulated in CRC. The high number of deregulated TFs is consistent with the dramatic transcriptional differences observed between cancer cells and normal epithelia (Birkenkamp-Demtroder *et al*, 2002; Kruhoffer *et al*, 2005). Among the 51 candidate TFs several were already known to be deregulated in CRC, for example *c-MYC* (He *et al*, 1998) and *SOX9* (Blache *et al*, 2004), and also many for which this information was novel. Of the latter, we selected four and validated their deregulation by qRT–PCR (E2F3) and/or IHC (E2F3, SOX4, CBFB and SMARCC1). For some of the TFs not previously reported to be deregulated in CRC, the involvement in the tumorigenesis of other cancers has been well established for example, *SOX4* in bladder cancer and *CBFB* in acute myeloid leukaemia (Kundu *et al*, 2002; Aaboe *et al*, 2006). Altogether, this indicates that many of the identified TFs are likely to play a role in the pathogenesis of CRC.

The RB/E2F pathway has been shown to be mutationally disrupted in many human cancers, but not in CRC (Nevins, 2001). It is still a matter of debate what role, if any, the RB/E2F pathway plays in the development of CRC (Hildebrandt *et al*, 2000; Nevins, 2001). Consistent with earlier studies not finding E2F3 in normal lung, bladder and prostate tissue (Feber *et al*, 2004; Foster *et al*, 2004; Cooper *et al*, 2006) we found that E2F3 was generally not expressed by the normal colon epithelia. In contrast, our analysis of CRC specimens showed high E2F3 transcript and protein levels, and revealed a dramatic reduction in the *RB1/E2F3* transcript ratio. We hypothesise that this reduction entails that the E2F inhibitor RB1 is titrated out and that the G1/S-transition checkpoint consequently is overridden, enabling the cancer cells to proliferate. We found no correlation between the E2F3 expression level and TNM stage or survival. This probably indicates that E2F3 is upregulated early in tumour development and hence not directly related to the outcome of the disease. Our data indicate that RB/E2F pathway inactivation may play an important role in CRC pathogenesis even though the mechanism of inactivation is different than in most other cancers.

To study if the 51 deregulated TFs were also involved in other aspects of CRC pathogenesis we investigated their transcript levels in the transcriptionally and clinically different MSS and MSI tumour subgroups. Twelve TFs were found to be differentially expressed between MSI and MSS tumours. However, compared with normal mucosa, 11 of these were deregulated in both MSS and MSI tumours making it difficult to judge if the differences between MSS and MSI have any biological consequences. The twelfth TF (*FOXA2*) was deregulated in the MSS tumours only. Hence, FOXA2 is likely to be regulating some of the many transcripts that are differentially expressed between MSS and MSI tumours.

We next investigated if the transcript levels of any of the selected 51 TFs were capable of predicting outcome of stage II CRC. The analyses indicated that three were putatively associated with recurrence of MSS stage II cancer and 13 putatively with recurrence of MSI stage II cancer. The higher number of candidates associated with recurrence of MSI cancer may be caused by a higher false-positive rate due to the limited number of samples in the MSI series (9 recurrent and 56 non-recurrent) compared with the MSS series (22 recurrent and 173 non-recurrent). The availability of transcriptional profiles from an independent cohort of stage II MSS cancer patients (Barrier *et al*, 2006) enabled us to investigate if any of the three MSS candidates could be validated. To our knowledge, no such data set is public available for MSI cancer; hence, the MSI candidates still await validation. Of the three MSS candidates only *SOX4* was also associated with recurrence in the independent cohort. In both cohorts, a high *SOX4* transcript level was significantly associated with recurrence of stage II MSS cancer and shorter recurrence-free survival. We only observed the association in MSS cancers consistent, with *SOX4* being differentially expressed between MSS and MSI tumours. By IHC analysis we demonstrated that the increased *SOX4* transcript levels, observed in CRC compared with normal mucosa, also translated to the protein level. Moreover, the IHC analysis revealed that the observed expression difference was cancer cell specific and not related to stromal changes. In summary, high *SOX4* transcript levels identify patients with a high risk of recurrence. These patients are likely to benefit from a more aggressive therapy than the standard treatment offered to stage II patients today. This study is not the first to link *SOX4* expression and clinical outcome of cancer. A recent study reported that the *SOX4* expression level correlated with survival in patients with urinary bladder cancer (Aaboe *et al*, 2006).

In normal cells, the transcriptional levels of TFs are tightly controlled by a network of signalling pathways. Several studies have shown that deregulation of pathways, for example, the K-ras/B-raf and Notch pathways, is a mechanism for deregulation of TFs in cancer (Sancho *et al*, 2004). We speculated whether pathway deregulation could be the mechanistic explanation for the altered expression patterns of the selected TFs. Mutational activation of the Wnt pathway occurs in 80–90% of all CRC cases and the pathway is thus a likely cause for deregulation of one or more of the TFs. Indeed, our array-based transcriptional profiling analysis of an inducible colon carcinoma Wnt-model system corroborated our hypothesis by indicating that 11 (∼20%) of the 51 TFs were Wnt-responsive genes. Importantly, these observations are supported by an earlier study that reported 5 of our 11 candidate Wnt-target TFs (*TGIF*, *SOX9*, *MYC*, *SOX4* and *TEAD4*) to be Wnt targets (Van der Flier *et al*, 2007). Collectively, our data indicate that deregulation of signalling pathways can lead to deregulation of TFs; this is likely to lead to deregulation of further signalling pathways, and in the end possibly to cellular transformation.

In the majority of CRC specimens we found the transcript and protein levels of both *CBFB* and *SMARCC1* increased compared with normal mucosa. Importantly, the observed differences were related to altered expression in the cancer cells and not the stroma cells. Univariate survival analysis revealed that patients with high protein levels of CBFB and SMARCC1 had a significantly longer overall survival rate than patients with low levels. Multivariate analysis further showed that the prognostic power of CBFB was independent of well-established prognostic factors, including TNM stage and differentiation grade. In keeping with the association between low levels of CBFB protein and poor survival, we observed that cases with negative/weak CBFB protein levels were significantly enriched in metastasis cases compared with primary adenocarcinomas. Our transcript analyses of *CBFB* and *SMARCC1* did not reveal any association, not even a trend, to recurrence of stage II cancer. We cannot explain why the protein but not the transcript level is associated with outcome. But it should be noted that the transcript analyses were performed using stage II cancers and time to recurrence data, whereas the protein analyses were performed using all TNM stages and overall survival data. This might in part explain the discrepancy. Consistent with earlier reports, we found CBFB protein both in the cytoplasm and the nucleus of the neoplastic cells (Tanaka *et al*, 1997). These locations are in agreement with the known function of CBFB, which constitutes half of the core-binding factor TF complex. Upon stimulation, cytoplasmic CBFB dimerises with the one of the CBF*α* factors (RUNX1–3), forming the core-binding factor complex, which in turn translocates to the nucleus and executes its TF function (Speck and Gilliland, 2002). Interestingly, *RUNX1* is one among the TFs that we found are upregulated. SMARCC1 is a core member of the SWI/SNF chromatin remodelling complex, and hence performs its function in the nucleus (Roberts and Orkin, 2004). In agreement with this, and with a previous report on the subcellular location of SMARCC1 in prostate cancer, we found SMARCC1 to be located in the nucleus (Heeboll *et al*, 2008). It is worth noting that in recent reports, the SWI/SNF chromatin remodelling complex, in line with our observations (SMARCC1), has become increasingly recognised for its role in tumour suppression (Roberts and Orkin, 2004; Nagl *et al*, 2007). To our knowledge, CBFB has not previously been associated with tumour suppressor activity. However, our data indicate that in CRC, possibly through interaction with RUNX1 and the formation of the core-binding factor complex, CBFB has this function. At first it may seem peculiar that high expression levels of genes, generally upregulated in tumours, can lead to prolonged patient survival; there are, however, mechanisms that could explain these findings. For example, as part of their natural defence mechanisms, normal cells will, when exposed to carcinogens, upregulate genes that suppress tumour development. Hence, during tumour development there will be selection towards clones that either simply downregulate these genes, or inactivate the signalling pathways through which they function. In cancers produced by the latter scenario, the tumour suppressor genes are upregulated but the cancer cells no longer respond to the signal. Genes involved in DNA damage response are examples of this (Bartkova *et al*, 2005).

In conclusion, we have identified 51 TFs that at the transcript level are deregulated in CRC compared with normal mucosa. Among these, *E2F3* is a key component of the RB/E2F G1/S transition checkpoint, indicating that inactivation of this checkpoint may be critical for the development of CRC. We showed that pathway deregulation, exemplified by the Wnt pathway, is a likely mechanism causing the observed TF deregulations. Furthermore, we found that the transcript levels of a subset of the TFs were associated with microsatellite status. For *E2F3*, *SOX4*, *CBFB* and *SMARCC1,* the transcript analyses were corroborated by an in-depth IHC analysis not only confirming their increased expression level, but also demonstrating their expression by the cancer cells. Finally, we showed that three of the identified TFs have prognostic potential. A high transcript level of *SOX4* predicts recurrence of stage II CRC, whereas high protein levels of SMARCC1 and CBFB predict long-term survival of CRC.

## Figures and Tables

**Table 1:** Fifty-one transcription factors deregulated in colorectal cancer

					**‘HG_U133A’ data set^a^**		**‘HG_U133 plus 2.0’ data set^b^**	
**Gene symbol**	**Entrez gene ID**	**Probe set ID^c^**	**Transfac ID^d^**	**Transcription actor class^d^**	***P*-value^e^**	**Fold change^f^**	***P*-value^e^**	**Fold change^f^**
*HAND1*	9421	220138_at	T04373	Basic helix–loop–helix	1.76E−05	−1.31	1.13E−09	−1.19
*HIS1*	10614	202815_s_at			9.84E−08	−1.15	4.22E−13	−1.67
*KLF4*	9314	220266_s_at		C2H2 zinc finger^g^	4.74E−05	−2.18	1.48E−09	−3.53
*MAF*	4094	206363_at		bZIP^g^	9.84E−08	−1.40	4.74E−22	−3.02
*MEF2C*	4208	209200_at	T01767	MADS box	1.17E−07	−1.96	1.36E−10	−2.86
*NR1H4*	9971	206340_at	T04498	Cys4 zinc finger of nuclear receptor type	2.40E−08	−2.70	4.17E−07	−1.92
*NR3C2*	4306	205259_at	T00513	Cys4 zinc finger of nuclear receptor type	3.52E−05	−2.20	7.39E−14	−4.02
*NR5A2*	2494	210174_at	T02771	Cys4 zinc finger of nuclear receptor type	1.37E−05	−1.66	4.39E−14	−3.56
*PPARGC1A*	10891	219195_at			5.49E−05	−1.14	2.15E−11	−2.66
*SLC26A3*	1811	215657_at			6.99E−08	−2.66	6.17E−14	−4.93
*SPIB*	6689	205861_at	T01401	Ets	1.92E−11	−2.71	4.08E−13	−2.03
*SSBP2*	23635	203787_at			1.93E−07	−1.23	1.29E−12	−2.47
*VDR*	7421	204254_s_at	T00885	Cys4 zinc finger of nuclear receptor type	2.43E−06	−1.20	4.03E−08	−1.36
*ZBTB16*	7704	205883_at		C2H2 zinc finger^g^	6.86E−08	−1.32	6.74E−13	−1.36
*ARNTL2*	56938	220658_s_at		Basic helix–loop–helix^g^	1.74E−05	1.58	2.28E−06	1.93
*BHLHB2*	8553	201170_s_at		Basic helix–loop–helix^g^	5.07E−08	1.96	1.29E−11	2.01
*CBFB*	865	202370_s_at	T02259	CBF_beta	2.12E−06	1.32	1.49E−13	1.49
*CBX4*	8535	206724_at			8.15E−05	1.11	9.62E−10	1.71
*CEBPB*	1051	212501_at		bZIP^g^	1.83E−05	1.00	6.84E−13	1.59
*DNMT1*	1786	201697_s_at		Zinc finger^g^	2.80E−05	1.18	7.61E−11	1.44
*E2F3*	1871	203693_s_at	T01545	Fork head/winged helix	2.46E−09	1.59	6.60E−14	1.69
*FOXA2*	3170	210103_s_at	T02413	Fork head	3.12E−07	2.06	2.75E−05	1.80
*FOXM1*	2305	202580_x_at	T02516	Fork head	1.17E−07	2.29	5.87E−15	3.10
*GTF2IRD1*	9569	218412_s_at		TFII-I^g^	3.18E−12	2.79	3.00E−22	2.77
*GTF3A*	2971	215091_s_at		C2H2 zinc finger^g^	3.54E−06	1.61	7.57E−13	2.00
*HIRA*	7290	217427_s_at			6.99E−08	1.43	2.61E−06	1.13
*HMGA1*	3159	206074_s_at		HMG^g^	1.91E−08	1.57	1.02E−19	2.50
*HMGB1*	3146	200679_x_at		HMG^g^	1.08E−05	1.26	5.54E−09	1.32
*MTA1*	9112	211783_s_at		Zinc finger^g^	6.96E−06	1.35	2.12E−11	1.57
*MYC*	4609	202431_s_at	T00140	bHLH-ZIP	4.10E−06	1.99	6.10E−14	2.66
*NFE2L3*	9603	204702_s_at		bZIP^g^	5.02E−07	1.80	5.15E−24	3.06
*NME2*	4831	201268_at	T00706		5.95E−09	1.08	2.39E−19	1.26
*NPM1*	4869	221923_s_at			5.22E−08	1.76	1.16E−12	1.59
*PHB*	5245	200658_s_at			5.01E−07	1.17	7.57E−10	1.25
*POLR3K*	51728	218866_s_at		Zinc finger^g^	8.15E−05	1.00	3.67E−06	1.12
*RUNX1*	861	209360_s_at	T01067	Runt	1.86E−05	1.30	3.34E−07	1.35
*SCML1*	6322	218793_s_at			1.32E−05	1.74	3.67E−07	1.68
*SMARCA4*	6597	213720_s_at		Bromo domain^g^	7.53E−07	1.11	2.42E−12	1.15
*SMARCC1*	6599	201074_at		Myb-like DNA-binding region^g^	8.84E−07	1.28	3.98E−11	1.15
*SOX4*	6659	213668_s_at	T01277	HMG	2.15E−07	2.84	1.06E−27	4.42
*SOX9*	6662	202935_s_at	T01853	HMG	1.38E−09	3.07	6.29E−27	3.91
*TEAD4*	7004	41037_at		TEA DNA-binding domain^g^	7.81E−10	2.24	2.99E−27	3.58
*TFDP1*	7027	212330_at	T01548	Fork head	7.04E−05	1.12	5.24E−07	1.21
*TGIF*	7050	203313_s_at	T04076	Homeo	1.17E−07	1.61	3.83E−20	2.44
*THRAP4*	9862	213043_s_at			2.51E−06	1.14	4.09E−15	1.59
*TRIB3*	57761	218145_at			1.66E−06	2.17	9.39E−20	3.73
*TRIM29*	23650	202504_at		Zinc finger^g^	6.99E−08	3.84	3.50E−11	4.39
*TRIP13*	9319	204033_at			2.40E−08	2.37	4.76E−23	3.32
*ZNF238*	10472	212774_at	T05040	C2H2-type zinc finger^g^	8.98E−07	1.38	6.55E−09	1.24
*ZNF263*	10127	203707_at		C2H2-type zinc finger^g^	3.97E−06	1.05	1.58E−16	1.46
*ZNF593*	51042	204175_at		C2H2-type zinc finger^g^	6.99E−08	1.45	3.32E−24	2.51

a The ‘HG_U133A’ data set consists of 10 normal mucosa and 80 colon cancer samples.

b The ‘HG_U133 plus 2.0’ data set consists of 10 normal mucosa and 344 colorectal cancer samples.

c Affymetrix GeneChip ‘HG_U133A’ and ‘HG_U133 plus 2.0’ probeset identifiers.

d According to TRANSFAC® 7.0 – Public (http://www.gene-regulation.com/pub/databases.html).

e Benjamini and Hockberg-corrected ‘Two Class Unpaired’ test for difference in expression level between normal mucosa and cancer samples.

f Fold change (on log 2 scale)=the average log value of the cancer samples subtracted the average log value of the normal mucosa samples.

g Characteristic transcription factor domains according to the Uniprot database (http://www.uniprot.org).

**Table 2:** Transcription factors associated with MSI status, recurrence of stage II CRC and Wnt signalling

**TF transcript level in**	**Up**	**Down**
MSI relative to MSS	*ARNTL2, HIS1*	*CBFB, FOXA2, GTF2IRD1, GTF3A, HMGB1, RUNX1, SMARCC1, SOX4, SOX9, ZNF238*
Recurrent relative to non-recurrent stage II MSS cancers	*SOX4, TRIM29*	*KLF4*
Recurrent relative to non-recurrent stage II MSI cancers	*MAF, KLF4, ZBTB16*	*FOXM1, GTF3A, HIS1, NPM1, PHB, POLR3K, SMARCA4, TRIB3, TRIP13, VDR*
Cells with inactive Wnt signalling relative to cells with active Wnt signalling	*BHLHB2, HIS1, SSBP2, TRIM29*	*ARNTL2, MYC, SMARCA4, SOX4, SOX9, TEAD4, TGIF*

CRC=colorectal cancer; MSS=microsatellite stabile; MSI=microsatellite instabile.

For detailed information on the sample sets investigated, the sizes of the fold changes observed and the statistical tests applied see Supplementary Tables 6–8.

**Table 3:** Univariate and multivariate analysis for overall survival (Cox proportional hazard regression model)

		**Univariate analysis**			**Multivariate analysis**		
**Factor**	**Patients (*n*=959)^a^**	**HR**	**95% CI**	***P-*value**	**HR**	**95% CI**	***P*-value**
*Age at surgery (years)*							
<65	317	1	(Reference)		1	(Reference)	
>65	642	1.69	1.38–2.06	<0.001	1.76	1.44–2.16	<0.001
							
*Sex (M/F)*							
Male	503	1	(Reference)		1	(Reference)	
Female	456	1.36	1.14–1.62	0.001	1.35	1.13–1.62	0.001
							
*Differentiation* b							
Well	13	1	(Reference)		1	(Reference)	
Moderate	821	3.02	0.97–9.41	0.056	1.89	0.60–5.99	0.276
Poor	116	5.05	1.59–16.04	0.006	2.9	0.90–9.38	0.074
							
*Tumour location*							
Right colon	332	1	(Reference)				
Left colon	302	0.81	0.65–1.00	0.053	—	—	—
Rectum	325	0.85	0.69–1.05	0.131	—	—	—
							
*Mucinous* b							
No	883	1	(Reference)				
Yes	70	1.01	0.85–1.20	0.89	—	—	—
							
*UICC TNM stage*							
I	128	1	(Reference)		1	(Reference)	
II	369	2.65	1.72–4.07	<0.001	2.33	1.51–3.60	<0.001
III	462	6.59	4.35–9.99	<0.001	5.88	3.87–8.95	<0.001
							
*SMARCC1 staining*							
Negative/weak	576	1	(Reference)		1	(Reference)	
Strong	383	0.82	0.68–0.98	0.029	0.95	0.78–1.14	0.57
							
*CBFB staining*							
Negative/weak	491	1	(Reference)		1	(Reference)	
Strong	468	0.72	0.61–0.86	<0.001	0.79	0.65–0.95	0.011

HR=hazard ratio; TF=transcription factor; 95% CI=95% confidence interval.

a To enable comparison of CBFB and SMARCC1, only patients with evaluable IHC staining for both TFs were included. Hence of the 1283 patients from the large custom-made TMA with available follow-up information, only 959 were included in the analysis.

b Patients with missing values were excluded from the analysis.
