# Supplementary figures and images for: Dysregulation of the transcription factors SOX4, CBFB and SMARCC1 correlates with outcome of colorectal cancer
Source: Br J Cancer. 2009 Jan 20;100(3):511–23. doi: 10.1038/sj.bjc.6604884 (PMC2658541; doi:10.1038/sj.bjc.6604884)

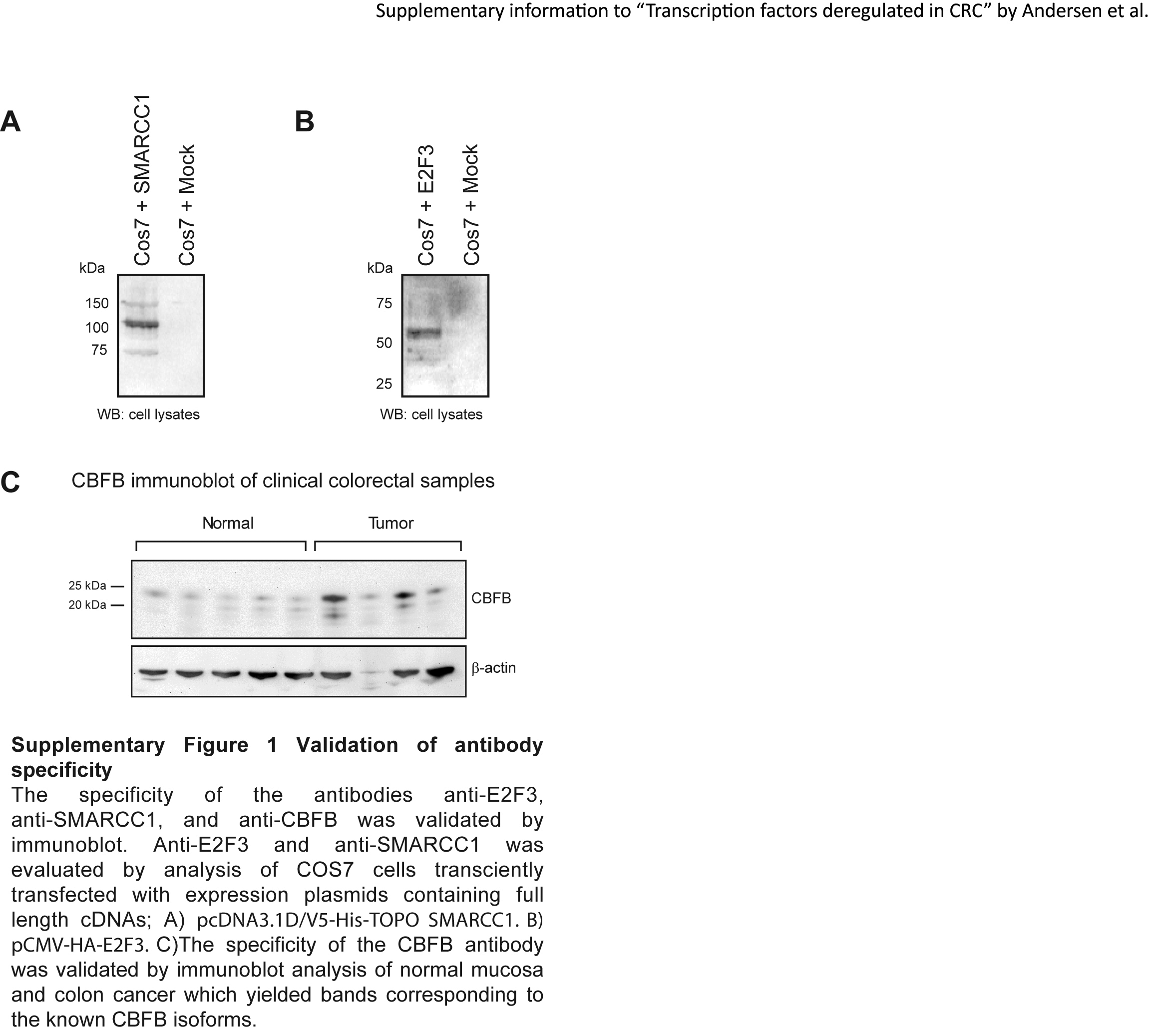

Supplement: Supplementary Figure 1 [file 6604884x1.tif]

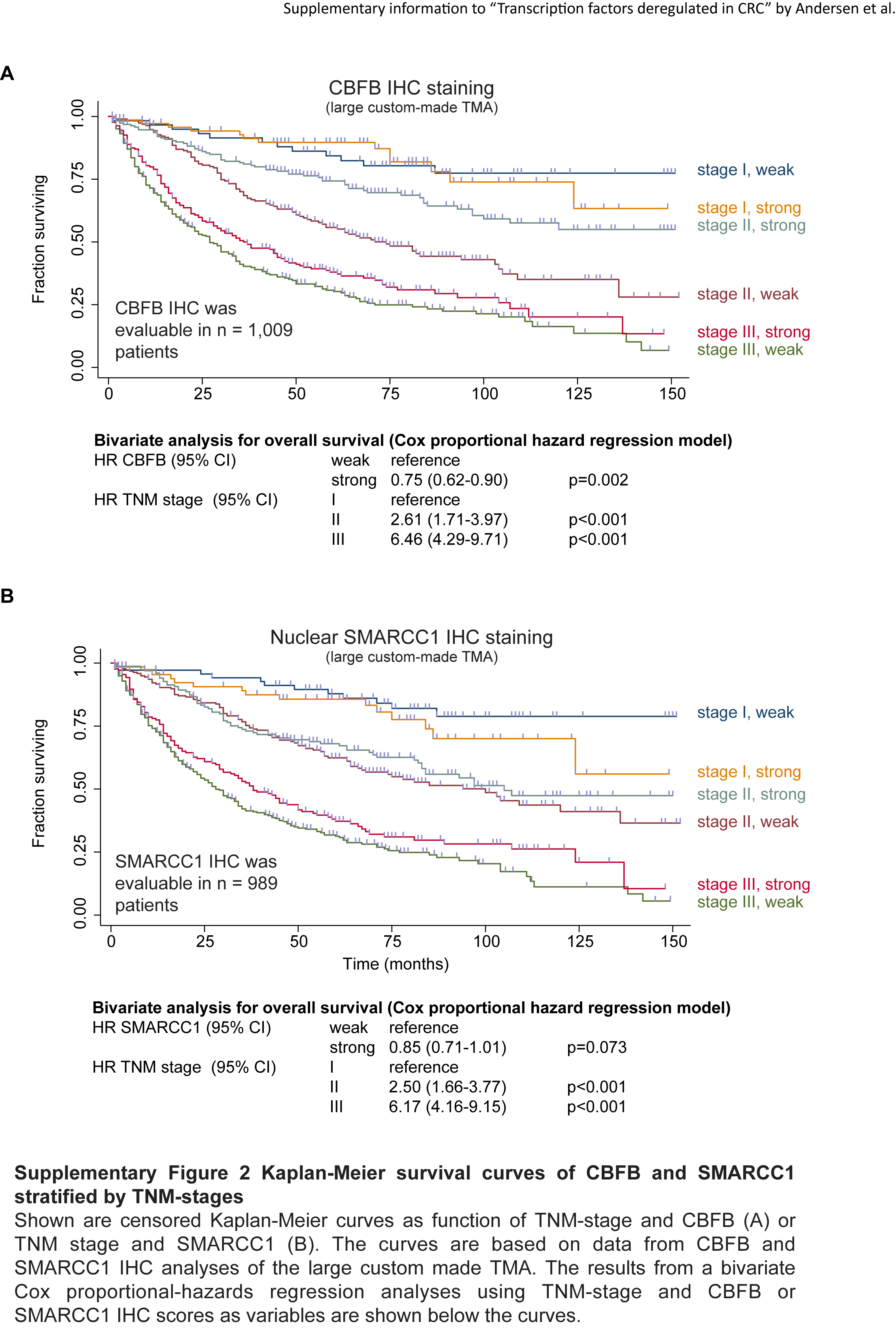

Supplement: Supplementary Figure 2 [file 6604884x2.tif]

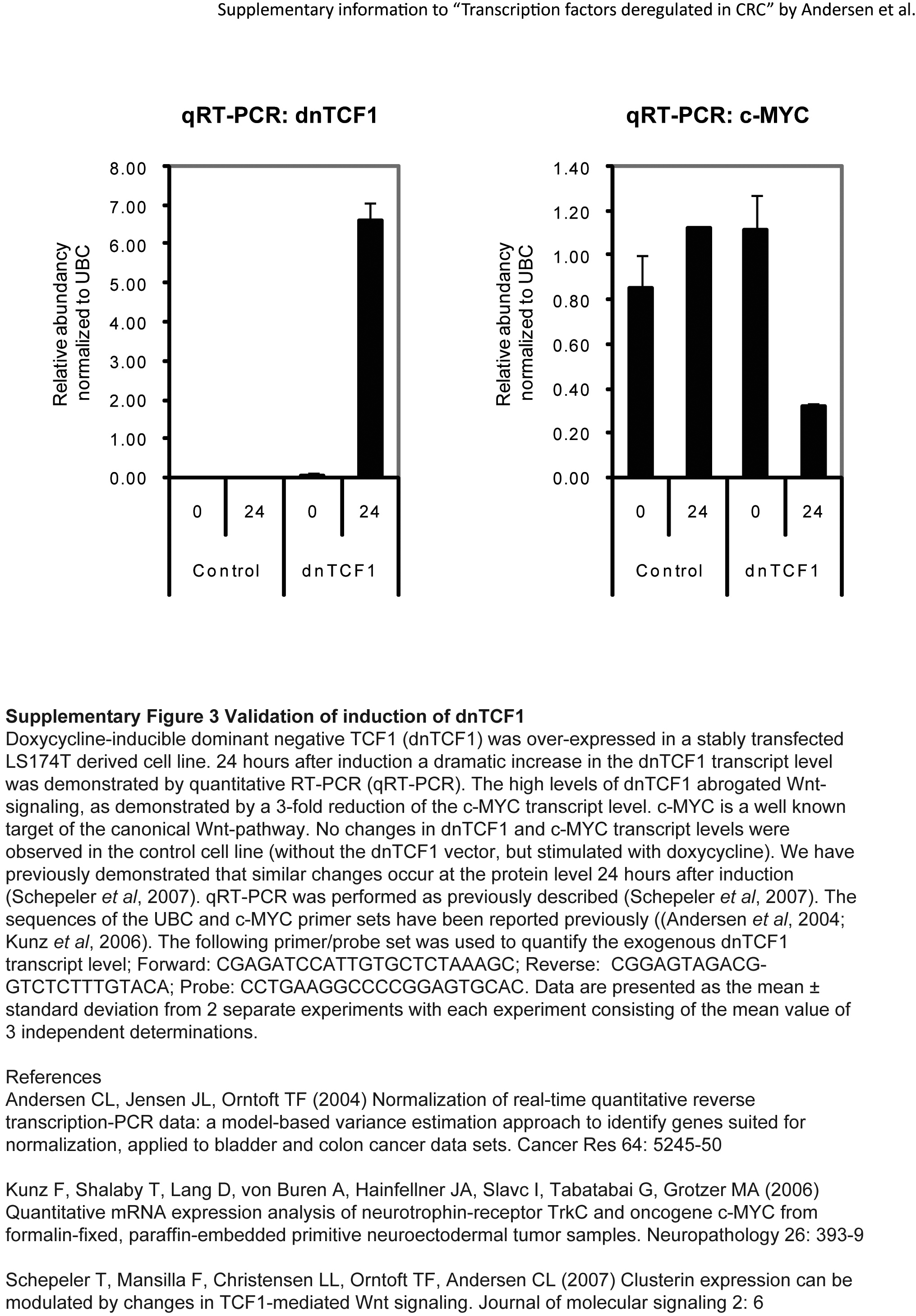

Supplement: Supplementary Figure 3 [file 6604884x3.tif]
