# Supplementary material for: Dysregulation of the transcription factors SOX4, CBFB and SMARCC1 correlates with outcome of colorectal cancer
Source: Br J Cancer. 2009 Jan 20;100(3):511–23. doi: 10.1038/sj.bjc.6604884 (PMC2658541; doi:10.1038/sj.bjc.6604884)
Supplement: Supplementary Information [file 6604884x5.doc]

# Supplementary information:

# Materials and methods for validation of antibodies used for immunohistochemistry

### Cell culture, plasmids, and transient transfection

Plasmids containing full length cDNA clones for E2F3 and SMARCC1 were kind gifts from Kristian Helin (pCMV-HA-E2F3) and Trevor Archer (pcDNA3.1D/V5-His-TOPO SMARCC1 (BAF155))(17). Empty (mock) and TF containing plasmids were transfected into COS7 cells using the Fugene 6 Transfection Reagent (Roche Diagnostics Corp., Indianapolis, IN) following the manufacturers instructions. COS7 cells were cultured in RPMI 1640 medium supplemented with 10% FCS and 1% penicillin-streptomycin. Twenty-four hours after transfection the COS7 cells were harvested and whole-cell protein extracts for immunoblotting were prepared by lysing cells in RIPA buffer with protease inhibitors (Roche).

### Immunoblotting

Whole cell extracts were prepared; pelleted cells were lysed on ice in a lysis buffer containing 50 mM Tris, pH 7.5, 150 mM NaCl, 1% NP-40, 0.5% deoxycholic acid supplemented with protease inhibitor cocktail (Roche Applied Science). Protein concentrations were determined by the Bradford assay. Samples were boiled 3 min in loading buffer (350 mM Tris HCL, 30% glycerol, 0.1% SDS, 600 mM DTT, 0.012 % W/V bromophenol blue) prior to loading an equal amount of protein (20 µg cell extract, 40 µg from medium) on each lane on precast NuPAGE 12% Bis-Tris Gel (Invitrogen). Electrophoresis and blotting onto a polyvinylidene fluoride (PVDF) membrane were performed according to standard minigel procedures for the Novex XCell II Mini-Cell system. All-Blue prestained standards (Bio-Rad) were used as molecular weight markers. After blocking with 3% skimmed milk, the membrane was incubated with primary antibodies; anti-CBFB (1:500), anti-SMARCC1 (1:1000), and anti-E2F3 (1:2000). Final protein detection was made using the secondary HRP-conjugated polyclonal goat anti-mouse antibody (1:2500; DakoCytomation, Glostrup, DK) in combination with ECL Plus reagent (Amersham Biosciences). As loading control, parallel immunoblotting using an anti-β-actin mouse monoclonal antibody (0.05 µg/ml, cat. no. A-1978, clone AC-15, Sigma-Aldrich A/S, Copenhagen, Denmark) was performed.
